# Circ-AKT3 inhibits clear cell renal cell carcinoma metastasis via altering miR-296-3p/E-cadherin signals

**DOI:** 10.1186/s12943-019-1072-5

**Published:** 2019-11-01

**Authors:** Dingwei Xue, Huan Wang, Yuanlei Chen, Danyang Shen, Jieyang Lu, Mingchao Wang, Abudureheman Zebibula, Liwei Xu, Haiyang Wu, Gonghui Li, Liqun Xia

**Affiliations:** Department of Urology, Sir Run Run Shaw Hospital, Zhejiang University School of Medicine, Hangzhou, 310016 China

**Keywords:** Circular RNAs, ccRCC metastasis, miRNA sponge, E-cadherin

## Abstract

**Background:**

Circular RNA (circRNA) is a type of circular endogenous RNA produced by special selective splicing and participates in progression of diverse diseases. However, the role of circRNA in clear cell renal cell carcinoma (ccRCC) is still rarely reported.

**Methods:**

We detected lower circ-AKT3 expression in ccRCC using the circular RNA microarray. Then, qPCR array was applied to verify the expression of circ-AKT3 in between 60 ccRCC tissues and adjacent normal tissues, as well as ccRCC cell lines and human normal kidney cell (HK-2). We investigated the function of circ-AKT3 in ccRCC in vitro and in vivo and detected underlying mechanisms by Western blotting, bioinformatic analysis, RNA pull-down assay and luciferase reporter assay.

**Results:**

Circ-AKT3 was verified significantly downregulated in ccRCC. Knockdown of circ-AKT3 promoted ccRCC migration and invasion, while overexpression of circ-AKT3 suppressed ccRCC metastasis. Further, circ-AKT3/miR-296-3p/E-cadherin axis was shown responsible for circ-AKT3 inhibiting ccRCC metastasis.

**Conclusion:**

Circ-AKT3 suppresses ccRCC metastasis by enforcing E-cadherin expression through competitively binding miR-296-3p. Circ-AKT3 may therefore serve as a novel therapeutic to better suppress ccRCC metastasis.

## Background

Renal cell carcinoma (RCC), one of the most common malignant cancers in the world, accounts for 4% of all newly diagnosed cancers and 2% of all cancer deaths in the United States [1]. Early stage or localized RCC is often treated with partial or radical nephrectomy, with a 5-year survival rate of 92.6%. However, about 30% of RCC patients are diagnosed at the metastatic stage, and those undergoing nephrectomy will also develop metastasis [2]. Though several drugs can be used to treat metastatic RCC, most patients experience cancer progression and eventually die. According to the histological classification, approximately 60–70% of RCC is clear cell renal cell carcinoma (ccRCC) [3]. Thus, to obtain a better treatment strategy for renal tumor patients, a more refined understanding of the mechanism of ccRCC metastasis is urgently needed

Circular RNAs (circRNAs) are formed as closed loops after precursor RNA splicing and are mostly generated from exons of protein-coding genes [4]. Owing to their circular structure, circRNAs have higher stability to exist in the human nuclei and avoid degradation by RNases [5]. With the frequent use of high throughput sequencing and in-depth mechanistic research, circRNAs have been found to have multiple functions. As one of the regulatory RNAs, circRNAs can promote gene transcription [6], regulate selective splicing [4], inhibit the maturation of miRNA [7], and promote protein-protein interactions as well as miRNA sponges [4, 8]. However, the role of circRNA in kidney disease, especially in ccRCC, is still unclear.

miRNAs are small non-coding RNAs, which are also responsible for the regulation of mRNA function, and a lot of miRNAs have been verified to play a pivotal role in the progression of renal cell carcinoma [9, 10]. The specific interaction between miRNAs and circRNAs that are involved in the metastatic renal cell carcinoma has not been fully discussed; therefore, insight into the effects of miRNAs and their potential circRNA regulators on metastasis progression may help to clarify the pathogenesis of ccRCC.

Emerging evidence suggests that cancer aggressiveness is associated with epithelial-mesenchymal transition (EMT) [11]. EMT contributes to promote tumorigenic progression of cancer cells with increased cell migration and invasion, ‘stemness,’ and inhibition of apoptosis and senescence [12–14]. The hallmark of EMT is the functional loss of cell adherent junctions [12]. The adherent junctions are mainly composed of a transmembrane calcium-dependent glycoprotein named E-cadherin (Gene ID: CDH1), which plays an important part in mechanical coupling of cells, thereby maintaining integrity both at local and tissue levels [15]. Besides, E-cadherin also participates in regulating cell signaling of cell death/ proliferation via the interaction of its cytoplasmic domain with the catenin family [16, 17]. Taken together, it is not surprising that E-cadherin was established as a tumor suppressor [18]. Moreover, its loss of function is associated with cancer invasion and metastasis [19, 20], thereby resulting in the poor prognosis of numerous carcinomas [21–23]. In ccRCC, a deregulation of E-cadherin expression is frequently observed in clinical samples [19] and loss of E-cadherin was regarded as an early oncogenic event [19, 24]. On the other hand, restoration of E-cadherin suppressed cancer progression including metastasis in various in vitro and in vivo tumor models [21, 26, 27]. However, the regulatory networks of E-cadherin by miRNA and circRNA in ccRCC remains elusive.

In this report, we identified a novel circRNA originating from the AKT3 gene locus, termed circ-AKT3, that was stably downregulated in ccRCC tissue samples and cell lines by microarray analysis and qPCR array. Functional assays showed that circ-AKT3 was negatively related to the metastasis of ccRCC cells. Mechanistic study revealed that circ-AKT3 may function as a sponge of miR-296-3p to upregulate E-cadherin expression, thus inhibiting ccRCC migration and invasion in vitro and metastasis in vivo*.* Circ-AKT3 may be applied as a therapeutic target to inhibit ccRCC metastasis.

## Materials and methods

### Human tissue specimens

Three pairs of snap-frozen ccRCC tissues and matched para-carcinoma normal tissues were obtained for circRNA microarray analysis, which were from patients who underwent partial nephrectomy at Department of Urology of Sir Run Run Shaw Hospital of School of Medicine of Zhejiang University (Hangzhou, China) between 2013 and 2018. Furthermore, sixty pairs of ccRCC tissues and paired adjacent normal kidney tissues were collected for validation. Histological and pathological diagnoses of the specimens were confirmed according to the 2016 World Health Organization Consensus Classification and Staging System of Renal Tumor and Fuhrman grade by two experienced pathologists. All specimens were obtained with appropriate informed consent of the patients and approved by the Ethics Committee of Sir Run Run Shaw Hospital, School of Medicine, Zhejiang University (IRB number: 20160222–20).

### Microarray analysis

circRNA microarray analysis was performed using Arraystar Human circRNA Array V2. Total RNA from each sample was quantified using the NanoDrop ND-1000. The sample preparation and microarray hybridization were performed according to the Arraystar’s standard protocols. Briefly, total RNAs samples were digested with Rnase R (Epicentre, Inc.) to exclude linear RNAs. Subsequently, the enriched circular RNAs were amplified and transcribed into fluorescent cRNA utilizing a random priming method (Arraystar Super RNA Labeling Kit; Arraystar). cRNAs were labeled and hybridized onto the Arraystar Human circRNA Array V2 (8x15K, Arraystar). After having washed the slides, the arrays were scanned by the Agilent Scanner G2505C. Agilent Feature Extraction software (version 11.0.1.1) was used to analyze the results. Quantile normalization and subsequent data processing was performed via the R software limma package. Normalized Intensity of each group (averaged normalized intensities of replicate samples, log2 transformed) were analyzed by paired t-test (*P* value cut off: 0.05). Differentially expressed circRNAs were identified through Fold Change filtering. Hierarchical Clustering was performed to show the distinguishable circRNAs expression pattern among samples.

### Cell lines and culture

Human normal kidney cell line HK-2 and ccRCC cell lines OSRC-2, Caki-1, SN12-PM6, A498, and SW839 were purchased from the American Type Culture Collection (ATCC, Manassas, VA, USA). All the cell lines were frozen in liquid nitrogen after the first 3 passages with 60 ampules of cell stock. After an ampule was thawed, cells were used within 15 passages in every designed experiment. OSRC-2-luciferase was established in previous study [25]. The cells were cultured in Dulbecco’s Modified Eagle’s Medium (Invitrogen, Grand Island, NY, USA) supplemented with 10% FBS (GIBCO, Brazil), penicillin (25 units/ml), streptomycin (25 g/ml), and 1% L-glutamine at 37 °C with 5% CO2.

### RNA extraction, reverse transcription, and quantitative real-time PCR analysis

Total RNAs were isolated using Trizol reagent (Takara Bio Inc., China). To verify the existence of circRNAs, 1 μg of total RNA was subjected to reverse transcription using Transcriptor First Strand cDNA Synthesis Kit ((Roche Diagnostics, Basel, Switzerland). The qRT-PCR was conducted using LightCycler 480 instrument (Roche Diagnostics) with SYBR green I (Roche Diagnostics) to determine the expression levels of circRNAs and mRNAs. Expression levels were normalized to the expression of GAPDH RNA. miRNA expression was detected using One Step PrimeScript miRNA cDNA Synthesis Kit (Takara Bio Inc., China). U6 acted as normalized controls. The relative fold-change in expression with respect to a control sample was calculated by the 2-ΔΔCt method. RNase R treatment was conducted as previously reported [26]. The primers were all listed in Additional file 1: Table S1.

### Wound healing assay

Cells were seeded into 6-well tissue culture plate until they reached ~ 70–80% confluence as a monolayer. The monolayer was gently and slowly scratched with a new 20 μL pipette tip across the center of the attached cells (0 h). After scratching, the wells were gently washed twice with phosphate-buffered saline (PBS) (Invitrogen, Grand Island, NY, USA) to remove the detached cells and residual serum. Subsequently, all the wells were refilled with fresh medium without serum and cells were incubated for additional 12 h and 24 h. Cell migration was photographed using microscopy (Primovert, Zeiss, Germany) and a 10× objective at the 12 h and 24 h after injury.

### Transwell assay

The invasive and migratory capacities of cells were evaluated via transwell assay. 2× 10^4/200 μL of cells were plated on the upper chambers with 8-μm pore filters (Millipore, Germany) coated with or without 50 μL Matrigel (BD Biosciences) in serum-free medium after 48 h post transfection of plasmid, DMEM with 10% FBS was added to the lower chambers as an attractant. After incubation for 12 h for migration and 48 h for invasion at 37 °C, non-migrating or non-invading cells were gently removed and cells migrated to the bottom of the membrane were fixed with 4% paraformaldehyde, stained with crystal violet solution for 30 min, and visualized under a microscope at × 100 magnification. All cells were counted in five randomly chosen microscopic fields.

### siRNA, plasmid construction and lentiviral transduction

Three siRNAs targeting circ-AKT3, and miRNA mimics (sequences were listed in Additional file 1: Table S1) were designed and synthesized by RiboBio (Guangzhou, China). The circ-AKT3-overexpressing lentivirus plasmid was synthesized by Geneseed (Guangzhou, China). Virus-containing supernatant was collected 48 h after lentivirus packaging, followed by its addition to the ccRCC cells (OSRC-2-luciferase, which stably expressed luciferase). After 24 h incubation, the stably infected cells were selected with 2 μg/mL of Puromycin 2HCL purchased from Selleck (Shanghai, China).

### RNA pulldown assay and AGO2-RIP assay

The RNA pulldown assay was performed as previously described with minor modification [27]. Biotin-labelled circ-AKT3 probe was synthesized by Tsingke (Beijing, China), the sequence of the probe was just complemented to the back-spliced junction of circ-AKT3(listed in Additional file 1: Table S1). Circ-AKT3 overexpressing OSRC-2 cells were scratched, lysed, and sonicated. After centrifugation, 50 μL of the supernatant was retained as input, and the remaining part was incubated with streptavidin dynabeads (M-280, Invitrogen) conjugated with biotin-labelled circ-AKT3 probe overnight at 4 °C. The mixture was washed, and total RNA was then isolated to detect the expression of circ-AKT3 and miRNAs by RT-PCR and qRT-PCR.

The AGO2-RIP assay was performed using the MagnaRIP RNA-Binding Protein Immunoprecipitation Kit (Millipore, MA, USA) as previously described [28]. After incubating circ-AKT3 overexpressing OSRC-2 cells lysate with dynabeads coated with 5 μg of control rabbit IgG or antibody against Argonaute-2 (AGO2) (Abcam, MA, USA) with rotation at 4 °C overnight, total RNA was retrieved for the detection of circ-AKT3 and miRNA expression by qRT-PCR.

### Luciferase report assay

Luciferase reporter vector pmirGLO with the full length of the 3′-UTR of E-cadherin or circ-AKT3 were constructed. Then we generated the mutant luciferase reporter vectors with QIAGEN XL-site directed Mutagenesis Kit (QIAGEN, California, USA). OSRC-2 and SW839 cells were seeded into 12-well plates and transfected with luciferase reporter vector and miR-296-3p mimics using the Lipofectamine 3000 reagent and Lipofectamine RNAiMAX reagent respectively. After 48 h incubation, the Firefly and Renilla luciferase activities were quantified with a dual-luciferase reporter assay (Promega, USA) according to the manufacturer’s protocol.

### Western blot analysis

Cells were lysed in RIPA buffer and proteins (30 μg) were separated on 8–12% SDS/PAGE gel and then transferred onto PVDF membranes (Millipore, Billerica, MA). After blocking membranes with non-fat milk for 1 h at room temperature, they were bred with proper dilutions of primary antibodies overnight at 4 °C. The following antibodies were used: monoclonal anti-GAPDH (Catalogue#5174, 1:5000), monoclonal anti-E-cadherin (Catalogue#3195, 1:1000), monoclonal anti-N-cadherin (Catalogue#13116, 1:1000), monoclonal anti-HIF-1α (Catalogue#36196, 1:1000), and monoclonal anti-Vimentin (Catalogue#5741, 1:1000) were all purchased from Cell signaling Technology; polyclonal anti-MMP2 (Catalog number: 10373–2-AP, 1:500) and polyclonal anti-MMP9 (Catalog number: 10375–2-AP, 1:500) were purchased from Proteintech; polyclonal anti-SMAD5 (ab88559, 1:1000) and polyclonal anti-STAT3 (ab119352, 1:1000) were purchased from Abcam; polyclonal anti-TIMP3 was purchased from ABGENT (70R-9697). Next day anti-mouse or anti-rabbit IgG secondary antibody (Cell signaling Technology, USA) were used for 1 h at the concentration of 1:5000 at room temperature and rinsed 10 min by TBST for 3 times. The bands were visualized using an ECL chemiluminescent detection system (Thermo Fisher Scientific, Rochester. NY).

### In vivo studies

Six to eight-week old nude mice were purchased and divided into 2 groups (*n* = 10) for injection stably infected ccRCC cells (Vector and OE-circ-AKT3) mixed with Matrigel (1:1) under subrenal capsule. Tumor development and metastasis were detected using in vivo imaging system (IVIS) after 8 weeks. Mice were sacrificed after 8 weeks, tumors and metastases were removed for studies. Studies on animals were conducted with approval from the Animal Research Ethics Committee of Zhejiang University (IACUC: ZJU20160141).

### Immunohistochemistry

Tumor tissues of 5 mice in each group were fixed in 10% (v/v) formaldehyde in PBS, embedded in paraffin, and cut into 5 μm sections and used for IHC staining with specific primary antibodies against E-cadherin (Catalogue#3195, Cell signaling technology). Positive degree was calculated as the number of immunopositive cells × 100% divided by total number of cells/field in 10 random fields at 200× magnification.

### Statistical analysis

Statistical analyses were performed using GraphPad Prism 6 (GraphPad Software, Inc., La Jolla, CA). group differences were tested for statistical significance using Student’s t-test, Kolmogorov-Siminov test, Mann–Whitney U-test, Wilcoxon matched-pairs signed rank test, Chi-square test and Spearman’s correlation, as appropriate. *P* < 0.05 was considered statistically significant.

## Results

### circRNA microarray analysis revealed the expression profile of circRNA in clear cell renal cell carcinoma

**Fig. 1:**
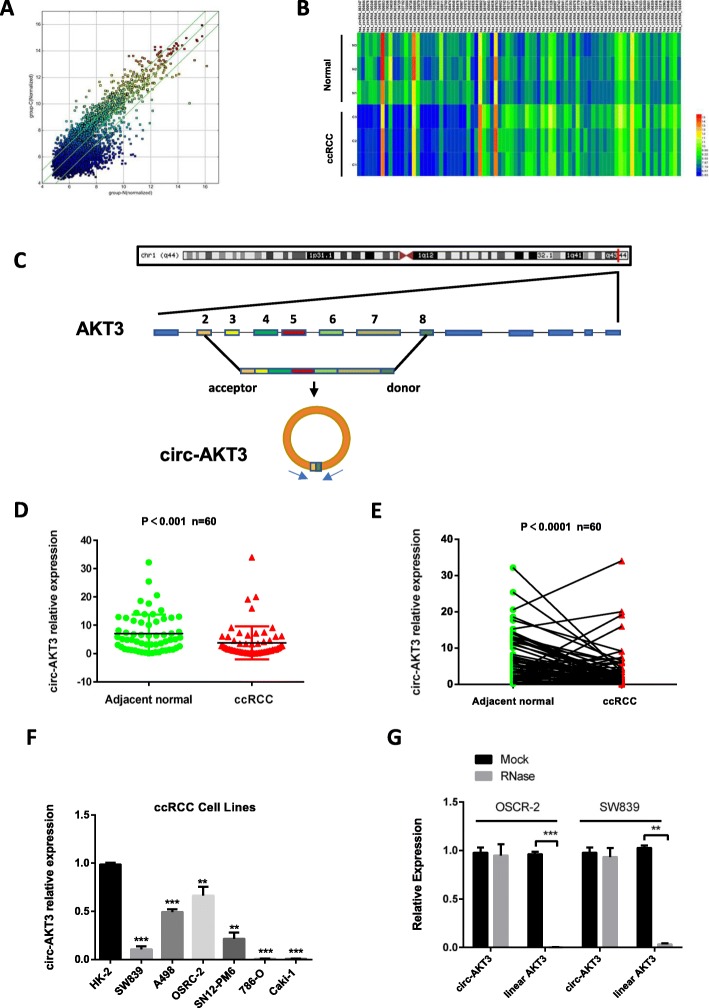
circRNAs expression profiles of ccRCC and characteristic of circ-AKT3. **a** Scatter plot compared the expression fold changes of circRNAs for ccRCC tissues versus adjacent normal kidney tissues. The green lines are Fold Change Lines. The circRNAs above the top green line and below the bottom green line indicated more than 2.0 fold change of circRNAs between the two compared groups. **b** Clustered heatmap for deregulated circRNAs in ccRCC (filtered by |FC (fold change)| ≥ 2 and *P* < 0.05), with rows representing tissues and columns representing circRNAs. The numerical data represented the circRNA in Arraystar. **c** Schematic illustration of circ-AKT3, the genomic structure shows that the sequence of circ-AKT3 contains seven exons (808 bp) from the AKT3 gene. Outward-facing primers (F/R) were designed against circ-AKT3 to examine its expression. **d** and **e** RT-qPCR analysis of expression fold change for circ-AKT3 in unpaired and paired ccRCC tissues compared with adjacent normal kidney tissues. **f** Expression level of circ-AKT3 in ccRCC cell lines were screened by qRT-PCR. OSRC-2, A498 and SM12-PN6 exhibited higher circ-AKT3 expression, while others are circ-AKT3-low-expressed. **g** The expression levels of circ-AKT3 and linear AKT3 mRNA were determined by qRT-PCR after RNase R treatment. Data are the means ± SEM of three independent experiments. **P < 0.01; ***P < 0.001

To investigate the circRNA expression profile in ccRCC, we analyzed 3 pairs of ccRCC tissue samples paired with para-carcinoma normal tissue using the circRNA microarray (Arraystar Human circRNA Array V2) (Fig. 1a). A total of 53 circRNAs was significantly up-regulated, and 30 circRNAs were down-regulated in ccRCC tissues (filtered by |FC (fold change)| ≥ 2 and *P* < 0.05) (Fig. 1b), which were listed in detail in Additional file 5: Table S4. These deregulations of circRNAs indicated their potential functions in ccRCC progression and demanded further investigation.

### Circ-AKT3 is stably low-expressed in ccRCC and is negatively correlated with the malignancy degree at diagnosis in ccRCC patients

We then chose the Top 10 most significantly high-expressed and low-expressed circRNAs to verify their abundance in ccRCC tissue samples and cell lines. First, we specially designed divergent primers to each selected circRNA. Interestingly, a circRNA (circRNA ID in circbase: hsa_circ_0017252, http://www.circbase.org/), which was derived from the AKT3 gene locus (Fig. 1c), was significantly decreased in the ccRCC tissues compared with normal kidney tissues, and this result was consistent with the circRNA microarray result (Fig. 1d and e). Further, circ-AKT3 was also found to be down-regulated in human ccRCC cell lines (OSRC-2, A498, SW839, 786-O, Caki-1, and SM12-PN6) compared with human renal tubular cells (HK-2) (Fig. 1f). In addition, the linear form (mRNA) of AKT3 was digested by RNase R, but circ-AKT3 was resistant to RNase R treatment (Fig. 1g), which indicates that the circ-AKT3 is in a circular form and it may function stably in cells.

On the other hand, we also analyzed the correlation of circ-AKT3 expression with clinicopathologic features of ccRCC patients and found that the expression level of circ-AKT3 significantly correlated with ccRCC Fuhrman grade which is generally used to predict ccRCC metastasis (Additional file 1: Table S3). This result indicated that the level of circ-AKT3 was negatively related to the degree of malignancy at diagnosis.

Together, results from Fig. 1d-f and Additional file 1: Table S3 demonstrate that circ-AKT3 is reduced in ccRCC tissues and cell lines and may be negatively correlated with degree of malignancy at diagnosis in ccRCC patients.

### Circ-AKT3 acts as a suppressor in the migration and invasion of ccRCC cells

**Fig. 2:**
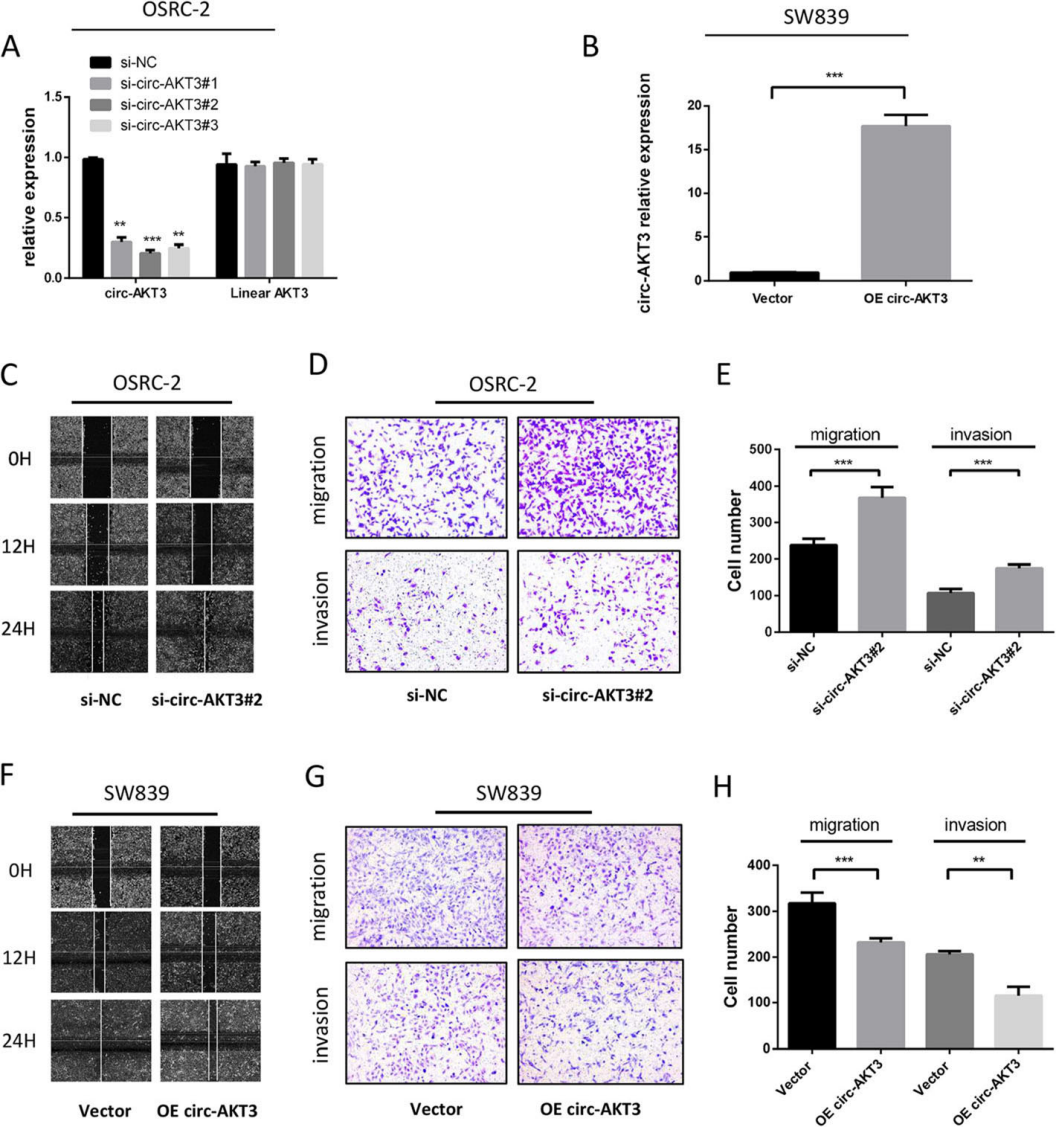
circ-AKT3 inhibits ccRCC cells migration and invasion in vitro. **a** Three siRNAs were designed to silence circ-AKT3 by targeting the spliced junction of circ-AKT3, it revealed that all the three siRNAs could downregulate the expression level of circ-AKT3 but had no effect on that of linear AKT3. Specially, si-circ-AKT3 #2 showed the highest silencing efficiency. **b** The plasmid over-expressing circ-AKT3 was constructed and its transfection efficiency was verified by qRT-PCR. **c** The cell migration capability was evaluated by the Wound healing assay in OSRC-2 cells transfected with si-Negative control (NC) and si-circ-AKT3 #2 respectively. **d-e** Transwell migration and invasion assays were performed to determine the role of circ-AKT3 in OSRC-2. **f** The cell migration capability was evaluated by the Wound healing assay in SW839 cells transfected with Vector and circ-AKT3-overexpressing plasmid respectively. **g-h** Transwell migration and invasion assays were performed to determine the role of circ-AKT3 in SW839. Data are the means ± SEM of three independent experiments. **P < 0.01; ***P < 0.001

To explore the role of circ-AKT3 in ccRCC, we used three siRNA oligonucleotides to target the unique back splicing junction of this circRNA. The antisense spliced junction siRNAs significantly decreased circ-AKT3 expression without decreasing the linear AKT3 mRNA level (Fig. 2a). Besides, circAKT3-overexpressing lentivirus plasmid was synthesized by Geneseed, and its transfection efficiency was detected by qRT-PCR (Fig. 2b). What’s more, AKT3 protein remained unchanged after altering the circ-AKT3 expression (Additional file 2: Figure S1). MTT assays revealed that circ-AKT3 does not affect the proliferation of ccRCC cells (Additional file 3: Figure S2A and B). However, after knockdown of circ-AKT3, OSRC-2 cells exhibited enhanced migratory capacity as demonstrated by the Wound healing assay, and vice versa (Fig. 2c). Consistently, the Transwell assay further demonstrated that the silencing of circ-AKT3 significantly promoted the migration and invasion of OSRC-2 cells, which contain relatively high endogenous circ-AKT3 (Fig. 2d and e). Meanwhile, overexpression of circ-AKT3 significantly inhibited the migration and invasion ability of SW839 cells which has relatively low endogenous circ-AKT3 (Fig. 2f-h). Taken together, results from Fig. 2c-h show that circ-AKT3 played a protective role through inhibiting migration and invasion in ccRCC cells.

### Circ-AKT3 inhibits ccRCC migration and invasion through restoring the expression of E-cadherin

**Fig. 3:**
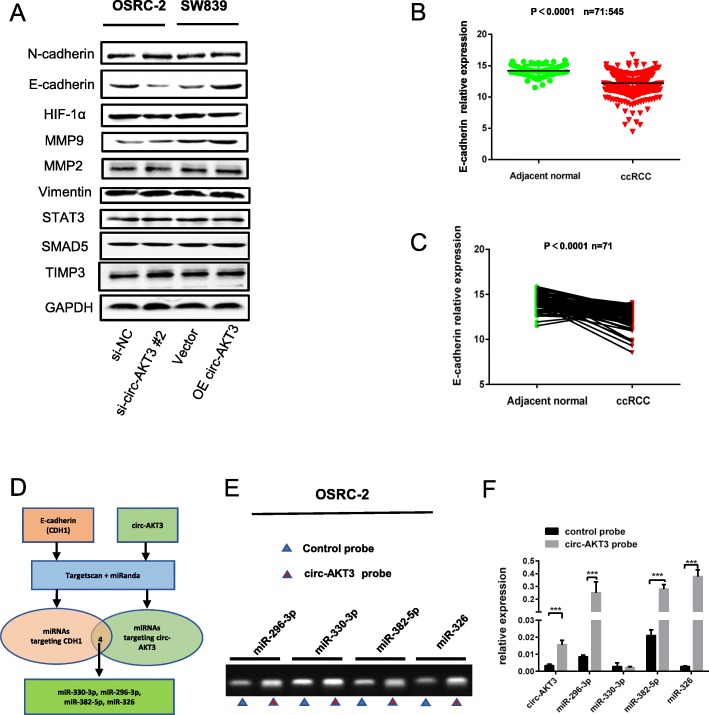
E-cadherin is involved in circ-AKT3-dependent inhibition of ccRCC cells migration and invasion. **a** Protein expression levels of metastasis related genes were screened through Western blotting analysis. **b-c** TCGA analysis of the expression levels of E-cadherin in paired and unpaired ccRCC tissues. **d** The schematic flowchart shows the pipelines of miRNAs which could bond to circ-AKT3 and E-cadherin 3′-UTR via online bioinformatic network. **e-f** the RT-PCR and qRT-PCR arrays determined that miR-296-3p, miR-382-5p and miR-326 could interact with circ-AKT3 via RNA pull down assay

With the purpose to detect the detailed mechanism of circ-AKT3 in ccRCC, we focused on a certain of genes involved in ccRCC metastasis to get some clues. Among them, E-cadherin, a metastasis related gene, is obviously regulated by circ-AKT3 as observed in the Western blotting result (Fig. 3a). Increasing evidences demonstrate that E-cadherin exerts a critically protective role in ccRCC metastasis [24, 29, 30]. Intriguingly, the mRNA levels of E-cadherin were mostly downregulated in tumoral tissues than in the matched nontumoral tissues based on an analysis of the TCGA database (Fig. 3b and c). E-cadherin expression was positively correlated with the circ-AKT3 expression level in ccRCC tissues (*p* < 0.005, r = 0.4004) (Additional file 4: Figure S3A). These data suggest that E-cadherin play a key role in mediating circ-AKT3-dependent inhibition of ccRCC migration and invasion.

### miR-296-3p is sponged by circ-AKT3 and plays a positive role in ccRCC metastasis

As circRNAs mostly function as miRNAs sponge to regulate downstream genes, we next explored the ability of circ-AKT3 to bind to miRNAs. Online bioinformatics databases (TargetScan and miRanda) were used to find potential miRNAs targeting E-cadherin mRNA and sponged by circ-AKT3, and 4 miRNAs were predicted (miR-330-3p, miR-296-3p, miR-382-5p, and miR-326) (Fig. 3d). Next, we performed RNA pulldown assay to test whether these miRNAs could bind to circ-AKT3 physically: circ-AKT3 probe, which is specifically antisense to the spliced junction sequence and conjugated with biotin, is designed to enrich circ-AKT3 and the miRNAs which it sponges (Fig. 3e). The relative expression of circ-AKT3 and miRNAs were analyzed by qRT-PCR (Fig. 3f). As is shown in Fig. 3e-f, miR-296-3p, miR-382-5p, and miR-326 were all proved to be sponged by circ-AKT3.

**Fig. 4:**
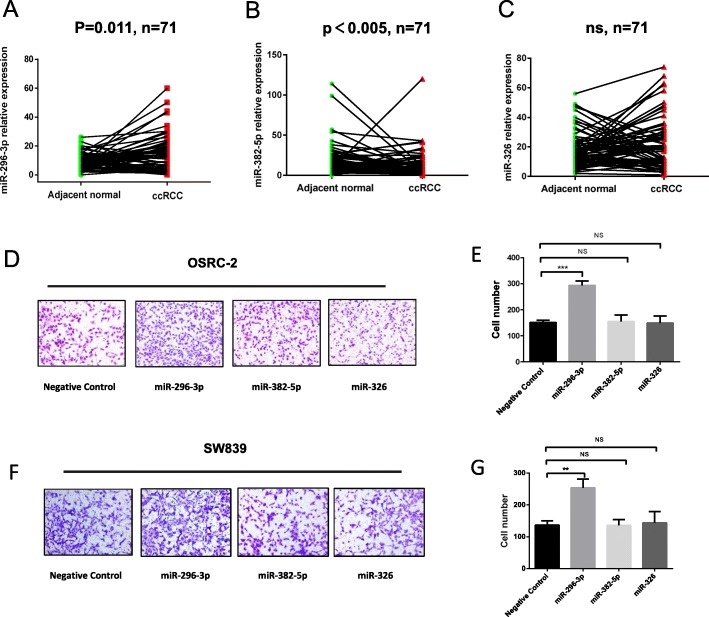
miR-296-3p is upregulated in ccRCC and promotes ccRCC cells metastasis. **a-c** TCGA analysis of the expression levels of miR-296-3p, miR-382-5p and miR-326 in paired ccRCC tissues. **d-g** Transwell assay was used to investigate the function of miR-296-3p, miR-382-5p and miR-326 in OSRC-2 and SW839 cells. Data are the means ± SEM of three independent experiments. **P < 0.01; ***P < 0.001

Subsequently, we analyzed the expression level of miR-296-3p, miR-382-5p and miR-326 in paired ccRCC tissues based on TCGA database. The results revealed that miR-296-3p expression was increased in paired and unpaired ccRCC tissues (Fig. 4a). Whereas, miR-382-5p showed decreased expression in ccRCC tissues (Fig. 4b) and miR-326 had no significant difference between ccRCC tissues and adjacent normal tissues (Fig. 4c). In addition, we also detected the expression level of miR-296-3p in our paired and unpaired ccRCC tissues, and it showed that miR-296-3p expression level was increased in ccRCC tissues (Additional file 4: Figure S3B). Besides, Spearman’s correlation analysis showed that miR-296-3p had a negative correlation with circ-AKT3 and E-cadherin expression (Additional file 4: Figure S3C and D). Further, upregulation of circ-AKT3 in both OSRC-2 and SW839 cells could significantly decrease the level of miR-296-3p (Additional file 4: Figure S3E). Transwell assay were applied to define the roles of miRNAs in ccRCC metastasis. Consistently, only miR-296-3p promoted ccRCC cells migration (Fig. 4d-g) while the other two miRNAs produced no difference in ccRCC cells migration.

As mentioned above, miR-296-3p was predicted to bind to circ-AKT3 as well as 3′ -untranslated region (3′-UTR) of E-cadherin mRNA. AGO2 is important in the regulation of “miRNAs Sponge” pathway, thus, RNA immunoprecipitation (RIP) for AGO2 (AGO2-RIP assay) in circ-AKT3 overexpressing OSRC-2 cells was then performed. It turned out that circ-AKT3 and miR-296-3p were substantially enriched by AGO2 (Additional file 4: Figure S3F).

Taken together, these results revealed that miR-296-3p played a positive role in the metastasis of ccRCC cells and circ-AKT3 serves as a sponge of miR-296-3p.

### miR-296-3p mediates circ-AKT3-dependent E-cadherin expression and cell migration and invasion

**Fig. 5:**
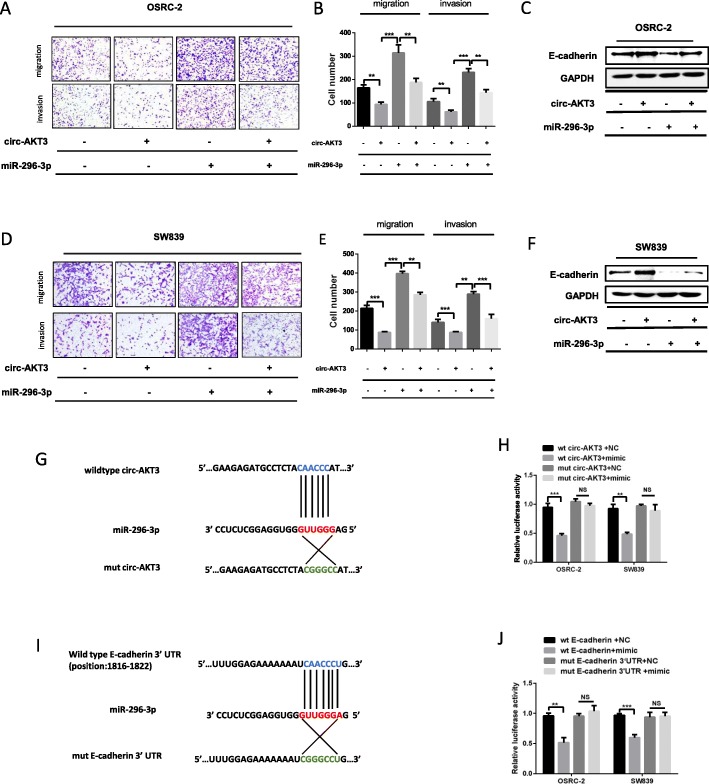
miR-296-3p mediates circ-AKT3-dependent E-cadherin expression and cell migration and invasion. **a-b** Transwell analysis after the co-transfection of miR-296-3p mimics and circ-AKT3 in OSRC-2 cells. **c** Western blotting analysis showed that miR-296-3p could decrease the protein level of E-cadherin and co-transfection of circ-AKT3 could reverse this effect in OSRC-2 cells. **d-f** Transwell and Western blotting analysis after the co-transfection of miR-296-3p mimics and circ-AKT3 in SW839 cells. **g-j** The luciferase reporter assay was used to detect the luciferase activity of pmirGLO-circAKT3wildtype/mutant and pmirGLO-E-cadherin-wildtype/mutant in OSRC-2 and SW839 cells co-transfected with miR-296-3p mimics, and the matched and mutant sequences were all shown. Data are the means ± SEM of three independent experiments. **P < 0.01; ***P < 0.001

To investigate the functional interaction between circ-AKT3 and miR-296-3p in ccRCC cells, we conducted the rescue experiment using Transwell assay. We found that, miR-296-3p mimics promoted the OSRC-2 cells migration and invasion, which could be rescued by addition of circ-AKT3 (Fig. 5a and b). Moreover, the miR-296-3p mimics significantly suppressed the E-cadherin expression, and this effect could be reversed after transfection with circ-AKT3-overexpresing plasmid (Fig. 5c). Transwell assay and western blotting analysis were also repeated in SW839 cells and the results were consistent with those of OSRC-2 cells (Fig. 5d-f).

To determine whether the predicted miR-296-3p binding site in circ-AKT3 sequence was essential for their interaction, the wild-type and mutated sequences of circ-AKT3 (in the predicted binding site) (Fig. 5g) were inserted into the luciferase reporter vector pmirGLO. Consequently, a dramatic decrease of luciferase activity was observed in OSRC-2 and SW839 cells transfected with the wild-type sequence, while those transfected with the mutant sequence showed no difference compared to the control (Fig. 5h). These data indicate that circ-AKT3 may serve as a sponge of miR-296-3p via the predicted binding site. Next, to determine whether miR-296-3p could bind to the E-cadherin mRNA 3′-UTR, we constructed the reporter plasmid with the wild-type E-cadherin 3′-UTR and the 3′-UTR with potential site mutation (Fig. 5i). As shown in Fig. 5j, miR-296-3p mimics could reduce the luciferase activity of cells transfected with the plasmid containing the wild type E-cadherin mRNA 3′-UTR sequence, whereas the effect was not shown in the group transfected with miR-296-3p binding site mutant, suggesting that miR-296-3p bind to the 3′-UTR of E-cadherin mRNA at the predicted binding site directly. Taken together, these results from Fig. 5a-j suggest that circ-AKT3 inhibits cell migration and invasion by acting as a sponge of miR-296-3p to avoid E-cadherin mRNA degradation to some extent.

### Restoration of circ-AKT3 suppresses ccRCC metastasis in vivo

**Fig. 6:**
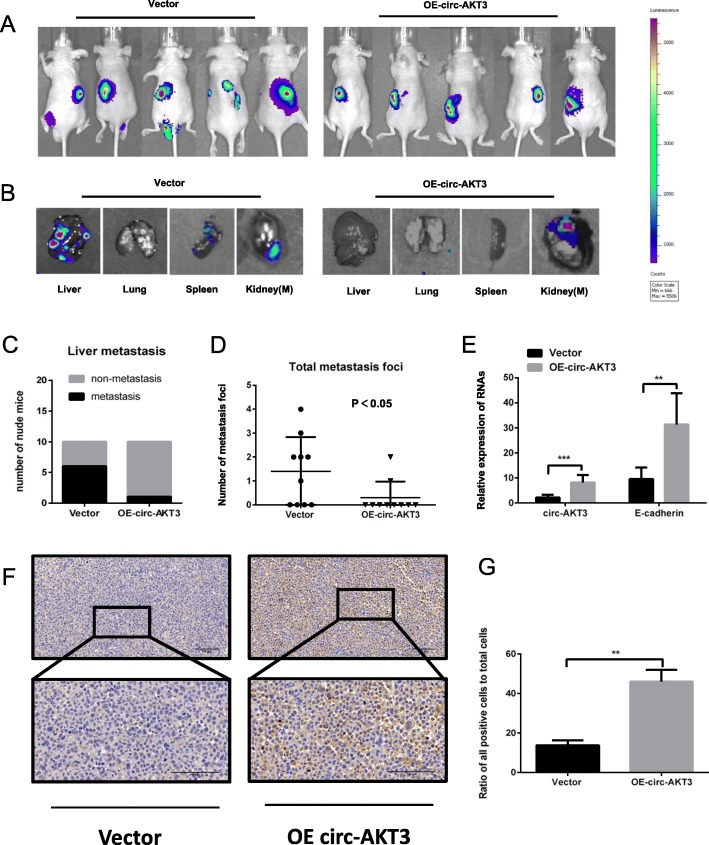
circ-AKT3 inhibits the metastasis of ccRCC in vivo. **a-b** Bioluminescence imaging of mice 8 weeks after the orthotopically implanted with OSRC-2-Luciferase-circ-AKT3 and OSRC-2-Luciferase-vector (*n* = 10 per group). Top panel: representative images. Bottom panel: images of metastatic foci of liver, lung, spleen and the metastatic kidney (Kidney(M)) on the other side. **c** Statistical analysis of mice with liver metastasis in each group. **d** Total metastatic foci of each mouse counted after dissection. **e** qRT-PCR analysis of circ-AKT3 and E-cadherin expression in ccRCC metastatic nodules. **f-g** Immunohistochemistry (IHC) detection of E-cadherin in tumor of mice. Scale bars: 100 μm. Three independent experiments were conducted. **P < 0.01; ***P < 0.001

Further in vivo investigation was made to determine the effects of enforced expression of circ-AKT3 on regulating ccRCC metastasis. OSRC-2 cells stably expressing luciferase (OSRC-2-luciferase) was infected with circ-AKT3 overexpressed lentivirus, after selection by puromycin, the OSRC-2-luciferase-circ-AKT3, or OSRC-2-luciferase-vector cells were injected under subrenal capsule of the BALB/c nude mice. As a result, nude mice injected with circ-AKT3 overexpressing cells (OE-circ-AKT3) formed fewer metastatic foci than those in vector group (Fig. 6a -d). Moreover, the level of E-cadherin expression level was obviously upregulated in OE-circ-AKT3 group compared with the control group (Fig. 6e). Consistently, Immunohistochemical staining (IHC) result showed that the E-cadherin expression was remarkably enhanced in the OE-circ-AKT3 tumors compared to tumors from the control group. (Fig. 6f and g). Taken together, these results demonstrate that enforced expression of circ-AKT3 efficiently inhibits the metastasis of ccRCC in vivo.

## Discussion

**Fig. 7:**
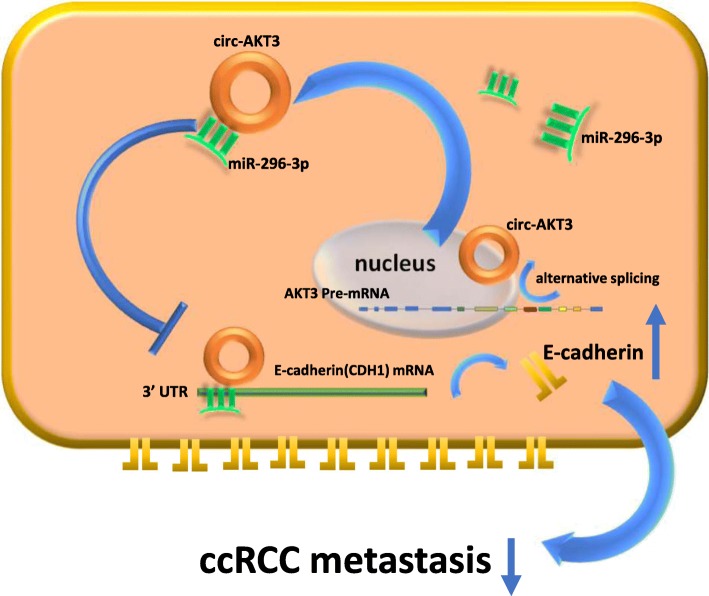
Schematic diagram illustrates the circ-AKT3 regulating pathway in ccRCC cells. Circ-AKT3 inhibits ccRCC metastasis by sponging miR-296-3p to restore E-cadherin expression

Currently, it is still a great challenge to treat metastatic RCC, thus the RCC metastasis mechanism has become a research focus. Recent studies have found several factors that could regulate the metastasis of renal cell carcinoma, such as VEGF [31], HIF-1α [32], HIF-2α [33], HGF [34], and E-cadherin [24, 29, 35], yet, how these factors are regulated remains unclear. Here, we found a new mechanism that the circ-AKT3 can go through the circAKT3/miR-296-3p/E-cadherin axis to suppress the ccRCC metastasis (Fig. 7). Our circRNA microarray and qRT-PCR data showed that circAKT3 was stably low-expressed both in the ccRCC tissue samples and cell lines. The in vitro and in vivo experiments revealed that circ-AKT3 acts as a sponge of miR-296-3p to upregulate E-cadherin, resulting in reduced migration and invasion of renal cell carcinoma.

CircRNA, a kind of stably expressed non-coding RNA, is arousing more and more interest, with accumulating reports showing that circRNAs are correlated with diverse diseases, specially the numerous types of cancer, such as hepatocellular cell carcinoma [27], lung cancer [36], breast cancer [37], and bladder cancer [38, 39]. Wang et al. found that circHIAT1 promotes the renal cell carcinoma via the regulation of androgen receptor (AR) [40]. Interestingly, in our study, it was clearly demonstrated that low expression of circ-AKT3 was a frequent event in ccRCC. Furthermore, in vitro and in vivo studies showed that upregulated expression of circ-AKT3 in ccRCC cells inhibited ccRCC cell migration and invasion.

To elucidate the detailed mechanism underlying circ-AKT3 function as a tumor suppressor in ccRCC, we first performed Western blotting assay to screen some metastasis related genes involved in the pathway of circ-AKT3-dependent inhibition of metastasis. Among the metastasis related genes, E-cadherin expression showed obvious and positive correlation with the expression level of circ-AKT3. It is notable that E-cadherin has been reported to act as a crucial suppressor of metastasis in most cancers, including ccRCC. Loss of E-cadherin is a vital event in the epithelial-to-mesenchymal transition of ccRCC, which is mainly due to HIF-1 activation as reported previously [24, 30]. Here, we verified for the first time that E-cadherin could be regulated by circRNAs.

Functional circRNAs were mostly reported to serve as a sponge for miRNAs, which participate in some specific signaling pathways in diseases. Until now, most of the studies on circRNAs were lacking systematic experimental verification, and mainly focused on bioinformatic analyses of all circRNA candidates. Importantly, we further determined whether circ-AKT3 can sponge certain miRNAs to rescue the function of downstream genes that inhibits the metastasis of ccRCC. Our data showed that circ-AKT3 contains the potential binding site of miR-296-3p, and it could bind to miR-296-3p in ccRCC cells as verified by RNA pulldown, AGO2-RIP, and Luciferase report assays. We therefore conclude that circ-AKT3 might function as a sponge of miR-296-3p.

In previous studies, miR-296-3p was reported to participate in the metastasis of numerous cancers [41–44]. It functions as an oncogene to promote migration and invasion in prostate cancer [31], but as a suppressor in lung adenocarcinoma metastasis [32]. However, its role in ccRCC was never studied. We determined that miR-296-3p promoted the migration and invasion of ccRCC cells, and this phenomenon could be reversed by circ-AKT3. Importantly, miR-296-3p was proven to reduce the expression of E-cadherin through binding to the 3′-UTR of E-cadherin using the Luciferase report assay and Western blotting analysis.

## Conclusion

In summary, our findings suggest a protective role of circ-AKT3 in the metastasis of ccRCC by sponging miR-296-3p and up-regulating E-cadherin expression. Therefore, circ-AKT3 may potentially be used in the future treatment against ccRCC, especially in patients with the metastatic ccRCC.

## Supplementary information

**Additional file 1: Table S1.** The sequences of primers and oligonucleotides used in this study. **Table S2.** Detailed information of ccRCC patients is listed. **Table S3.** Correlation of circ-AKT3 expression with clinicopathologic features of ccRCC patients.

**Additional file 2: Figure S1.** (A)AKT3 protein level after altering circ-AKT3 in OSRC-2 and SW839.

**Additional file 3: Figure S2.** (A-B). MTT assay indicated circ-AKT3 makes no difference in the proliferation of ccRCC cell lines. Data are the means ± SEM of three independent experiments.

**Additional file 4: Figure S3.** (A) Correlations were identified between circ-AKT3 and E-cadherin expression level in 60 paired ccRCC tissues. (B) RT-qPCR analysis of miR-296-3p expression in our own ccRCC tissues. (C) Correlations analysis between circ-AKT3 and miR-296-3p expression level in 60 paired ccRCC tissues. (D) Correlations analysis between E-cadherin and miR-296-3p expression level in 60 paired ccRCC tissues. (E) miR-296-3p expression level was decreased after altering circ-AKT3 in ccRCC cell lines. (F) AGO2-RIP assay was conducted to further verify that circ-AKT3 and miR-296-3p coexisted in AGO2 pellet to affect downstream gene post-transcription. Data are the means ± SEM of three independent experiments. *P<0.05, **P < 0.01; ***P < 0.001.

**Additional file 5: Table S4.** Differently expressed circRNAs expression profiles in ccRCC via microarray.

## Data Availability

The datasets supporting the conclusions of this article are included within the article and its additional files.

## Abbreviations

3′-UTR: 3′ -untranslated region
AGO2-RIP: AGO2 RNA-immunoprecipitation
AKT3: AKT serine/threonine kinase 3
AR: Androgen receptor
ccRCC: Clear cell renal cell carcinoma
CDH1: E-cadherin
circRNA: Circular RNA
EMT: Epithelial-mesenchymal transition
HGF: Hepatocyte growth factor
HIF: Hypoxia-Inducible Factor
IHC: Immunohistochemical staining
miRNA: MicroRNA
PBS: Phosphate-buffered saline
qRT-PCR: Quantitative real-time PCR
TCGA: The cancer genome atlas
VEGF: Vascular endothelial growth factor
