# Supplementary material for: Circ-AKT3 inhibits clear cell renal cell carcinoma metastasis via altering miR-296-3p/E-cadherin signals
Source: Mol Cancer. 2019 Nov 1;18:151. doi: 10.1186/s12943-019-1072-5 (PMC6824104; doi:10.1186/s12943-019-1072-5)
Supplement: Supplementary file 1 — Additional file 1: Table S1. The sequences of primers and oligonucleotides used in this study. Table S2. Detailed information of ccRCC patients is listed. Table S3. Correlation of circ-AKT3 expression with clinicopathologic features of ccRCC patients. [file 12943_2019_1072_MOESM1_ESM.docx]

**Additional file 1:**

**Table S1. The sequences of primers and oligonucleotides used in this study**

| **Primers** | |
| --- | --- |
| circ-AKT3 F | TCCTTCCAGACAAAAGACCGT |
| circ-AKT3 R | CGCTCATGATGACTCCCCTC |
| Linear AKT3 F | CCTTGGTGGAGGACCAGATG |
| Linear AKT3 R | TTGCTGACATTTTTCAGGTGG |
| GAPDH F | GTCAAGGCTGAGAACGGGAA |
| GAPDH R | AAATGAGCCCCAGCCTTCTC |
| CDH1(E-cadherin) F | GTCTGTAGGAAGGCACAGCC |
| CDH1(E-cadherin) R | TCATCCTCTGGGGGCAGTAA |
| Hsa-miR-330-3p | GCAAAGCACACGGCCTGCAGAGA |
| Hsa-miR-296-3p | GAGGGTTGGGTGGAGGCTCTCC |
| Hsa-miR-382-5p | GAAGTTGTTCGTGGTGGATTCG |
| Hsa-miR-326 | CCTCTGGGCCCTTCCTCCAG |
| U6 F | CTCGCTTCGGCAGCACA |
| U6 R | AACGCTTCACGAATTTGCGT |
| miR reverse | One Step PrimeScript miRNA cDNA Synthesis Kit, TaKaRa |
| **siRNAs Targeting sequence** | |
| Si circ-AKT3 1# | CGAGGCTGAGTCATCACTA |
| Si circ-AKT3 2# | GCGAGGCTGAGTCATCACT |
| Si circ-AKT3 3# | GGGCGAGGCTGAGTCATCA |
| **miRNAs mimics** | |
| Hsa-miR-330-3p sense | GCAAAGCACACGGCCUGCAGAGA |
| Hsa-miR-330-3p anti-sense | CGUUUCGUGUGCCGGACGUCUCU |
| Hsa-miR-296-3p sense | GAGGGUUGGGUGGAGGCUCUCC |
| Hsa-miR-296-3p anti-sense | CUCCCAACCCACCUCCGAGAGG |
| Hsa-miR-382-5p sense | GAAGUUGUUCGUGGUGGAUUCG |
| Hsa-miR-382-5p anti-sense | CUUCAACAAGCACCACCUAAGC |
| Hsa-miR-326 sense | CCUCUGGGCCCUUCCUCCAG |
| Hsa-miR-326 anti-sense | GGAGACCCGGGAAGGAGGUC |
| **Biotinylated probes** | |
| Biotin-circ-AKT3 | CAATTACCCCCGCTCCGACTCAGTAGTGATCT |

**Table S2. Detailed information of ccRCC patients is listed**

| Patient number | Age at surgery | Gender | T | N | M | Pathological stage | Fuhrman grade |
| --- | --- | --- | --- | --- | --- | --- | --- |
| 1 | 48 | Male | 1b | 0 | 0 | I | 2 |
| 2 | 56 | Male | 3a | 0 | 0 | III | 3 |
| 3 | 84 | Male | 1b | 0 | 0 | I | 2 |
| 4 | 54 | Male | 2a | 0 | 0 | II | 3 |
| 5 | 81 | Male | 1a | 0 | 0 | I | 3 |
| 6 | 76 | Male | 1a | 0 | 0 | I | 3 |
| 7 | 50 | Male | 1a | 0 | 0 | I | 2 |
| 8 | 71 | Male | 1b | 0 | 0 | I | 3 |
| 9 | 59 | Male | 1a | 0 | 0 | I | 2 |
| 10 | 55 | Male | 2a | 0 | 0 | II | 3 |
| 11 | 75 | Female | 1b | 0 | 0 | I | 2 |
| 12 | 72 | Male | 1a | 0 | 0 | I | 3 |
| 13 | 73 | Female | 1b | 0 | 0 | I | 2 |
| 14 | 60 | Male | 1a | 0 | 0 | I | 3 |
| 15 | 37 | Male | 1a | 0 | 0 | I | 2 |
| 16 | 70 | Male | 1a | 0 | 0 | I | 3 |
| 17 | 41 | Male | 2b | 0 | 0 | II | 2 |
| 18 | 57 | Male | 1a | 0 | 0 | I | 3 |
| 19 | 60 | Male | 1a | 0 | 0 | I | 3 |
| 20 | 45 | Female | 2a | 0 | 0 | II | 3 |
| 21 | 42 | Male | 1a | 0 | 0 | I | 3 |
| 22 | 54 | Male | 1b | 0 | 0 | I | 2 |
| 23 | 49 | Male | 1a | 0 | 0 | I | 2 |
| 24 | 56 | Female | 2a | 0 | 0 | II | 2 |
| 25 | 70 | Female | 1b | 0 | 0 | I | 2 |
| 26 | 61 | Female | 1b | 0 | 0 | I | 2 |
| 27 | 69 | Male | 1b | 0 | 0 | I | 2 |
| 28 | 32 | Male | 1a | 0 | 0 | I | 2 |
| 29 | 81 | Male | 1b | 0 | 0 | I | 2 |
| 30 | 52 | Female | 1a | 0 | 0 | I | 2 |
| 31 | 79 | Male | 1a | 0 | 0 | I | 3 |
| 32 | 31 | Male | 1b | 0 | 0 | I | 2 |
| 33 | 85 | Female | 1b | 0 | 0 | I | 2 |
| 34 | 56 | Male | 1a | 0 | 0 | I | 2 |
| 35 | 46 | Male | 1a | 0 | 0 | I | 2 |
| 36 | 55 | Female | 1a | 0 | 0 | I | 2 |
| 37 | 70 | Male | 1a | 0 | 0 | I | 2 |
| 38 | 79 | Female | 1b | 0 | 0 | I | 2 |
| 39 | 63 | Male | 1b | 0 | 0 | I | 2 |
| 40 | 56 | Male | 1a | 0 | 0 | I | 2 |
| 41 | 37 | Male | 1b | 0 | 0 | I | 2 |
| 42 | 72 | Male | 1b | 0 | 0 | I | 2 |
| 43 | 40 | Male | 1a | 0 | 0 | I | 2 |
| 44 | 64 | Male | 2a | 0 | 0 | II | 2 |
| 45 | 52 | Female | 1a | 0 | 0 | I | 2 |
| 46 | 58 | Male | 1a | 0 | 0 | I | 2 |
| 47 | 57 | Male | 1a | 0 | 0 | I | 2 |
| 48 | 60 | Male | 1a | 0 | 0 | I | 3 |
| 49 | 73 | Female | 1b | 0 | 0 | I | 2 |
| 50 | 51 | Female | 1a | 0 | 0 | I | 2 |
| 51 | 51 | Female | 2a | 0 | 0 | II | 2 |
| 52 | 56 | Female | 1a | 0 | 0 | I | 2 |
| 53 | 52 | Male | 2b | 0 | 0 | II | 2 |
| 54 | 73 | Female | 1b | 0 | 0 | I | 2 |
| 55 | 44 | Male | 1b | 0 | 0 | I | 1 |
| 56 | 67 | Male | 1a | 0 | 0 | I | 2 |
| 57 | 56 | Female | 1b | 0 | 0 | I | 1 |
| 58 | 56 | Male | 1a | 0 | 0 | I | 1 |
| 59 | 59 | Male | 1a | 0 | 0 | I | 2 |
| 60 | 29 | Male | 1a | 0 | 0 | I | 2 |

**Table S3. Correlation of circ-AKT3 expression with clinicopathologic features of ccRCC patients**

Parameter Group circ-AKT3 expression level P value

Cases Low High

Gender Male 43 23 20 0.3901

Female 17 7 10

Age at surgery ≥55 39 19 20 0.7866

＜55 21 11 10

T grade 1a+1b 51 24 27 0.2781

2a+2b+3a 9 6 3

Pathological stage I 51 24 27 0.2781

II+III 9 6 3

Fuhrman grade 1+2 46 19 27 0.0303*

3 14 11 3
