# Supplementary figures and images for: Circ-AKT3 inhibits clear cell renal cell carcinoma metastasis via altering miR-296-3p/E-cadherin signals
Source: Mol Cancer. 2019 Nov 1;18:151. doi: 10.1186/s12943-019-1072-5 (PMC6824104; doi:10.1186/s12943-019-1072-5)

A

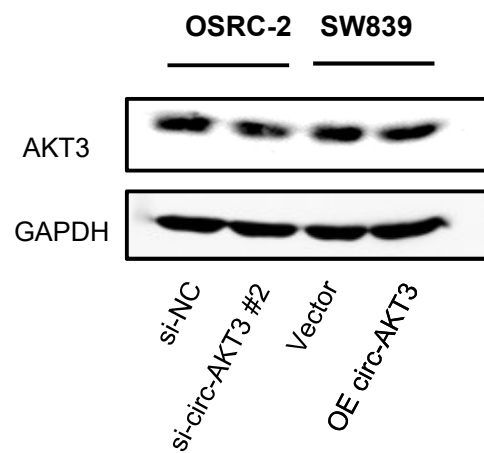

Supplement: Supplementary file 2 — Additional file 2: Figure S1. (A)AKT3 protein level after altering circ-AKT3 in OSRC-2 and SW839. [file 12943_2019_1072_MOESM2_ESM.pdf]

**A**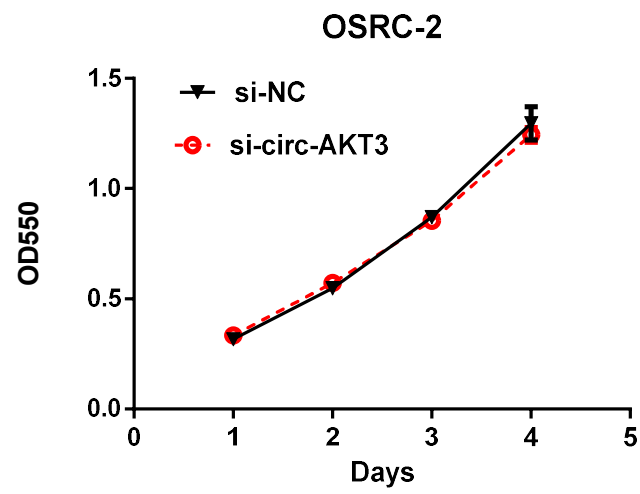**B**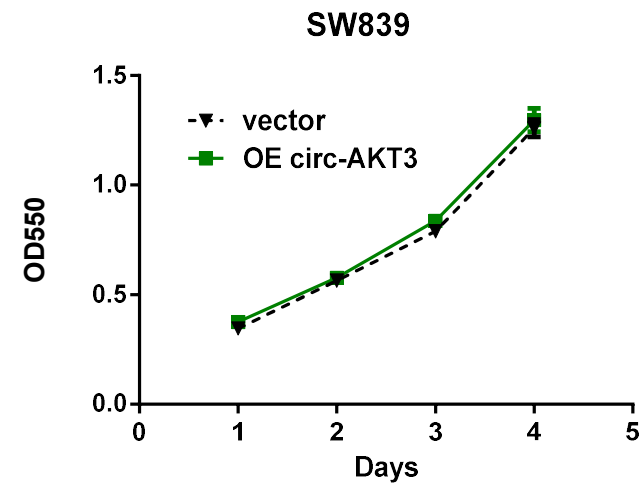

Supplement: Supplementary file 3 — Additional file 3: Figure S2. (A-B). MTT assay indicated circ-AKT3 makes no difference in the proliferation of ccRCC cell lines. Data are the means ± SEM of three independent experiments. [file 12943_2019_1072_MOESM3_ESM.pdf]

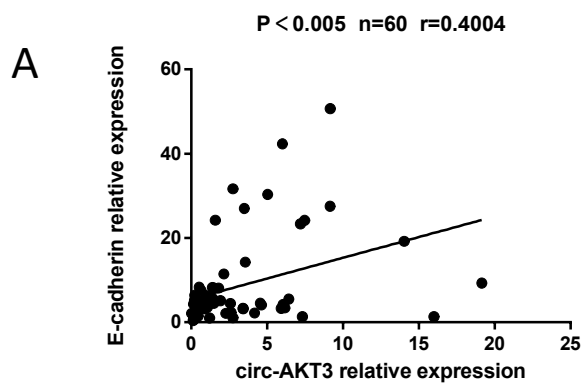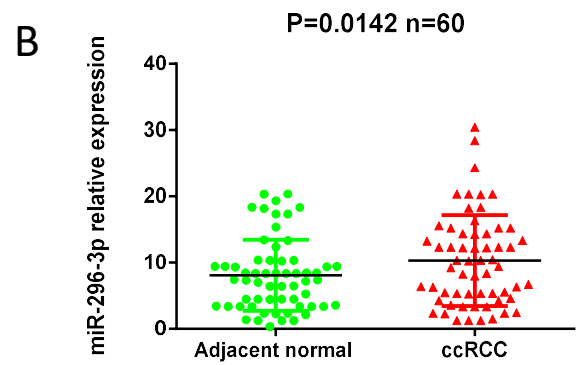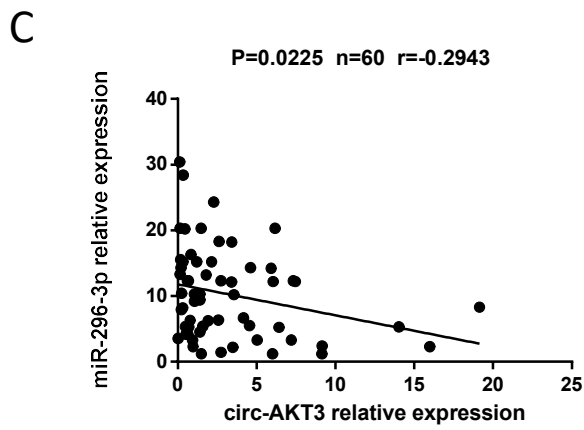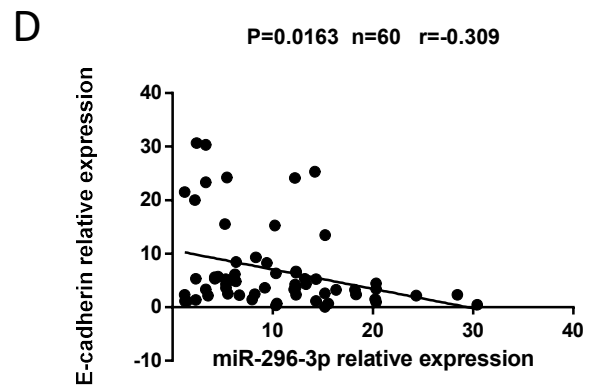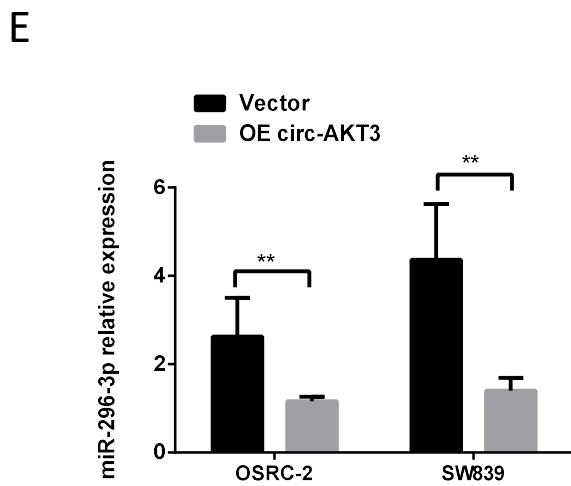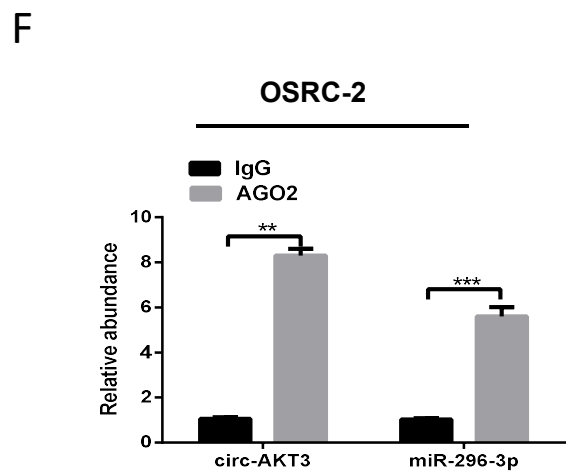

Supplement: Supplementary file 4 — Additional file 4: Figure S3. (A) Correlations were identified between circ-AKT3 and E-cadherin expression level in 60 paired ccRCC tissues. (B) RT-qPCR analysis of miR-296-3p expression in our own ccRCC tissues. (C) Correlations analysis between circ-AKT3 and miR-296-3p expression level in 60 paired ccRCC tissues. (D) Correlations analysis between E-cadherin and miR-296-3p expression level in 60 paired ccRCC tissues. (E) miR-296-3p expression level was decreased after altering circ-AKT3 in ccRCC cell lines. (F) AGO2-RIP assay was conducted to further verify that circ-AKT3 and miR-296-3p coexisted in AGO2 pellet to affect downstream gene post-transcription. Data are the means ± SEM of three independent experiments. *P<0.05, **P < 0.01; ***P < 0.001. [file 12943_2019_1072_MOESM4_ESM.pdf]
